# Correlates of parasites and pseudoparasites in wolves (*Canis lupus*) across continents: A comparison among Yellowstone (USA), Abruzzo (IT) and Mercantour (FR) national parks

**DOI:** 10.1016/j.ijppaw.2019.09.002

**Published:** 2019-09-12

**Authors:** Barbara Molnar, Paolo Ciucci, Gianluca Mastrantonio, Bruno Betschart

**Affiliations:** a Institute of Biology, University of Neuchâtel, Rue Emile-Argand 11, CH-2000, Neuchâtel, Switzerland; b Department of Biology and Biotechnologies “Charles Darwin”, Sapienza University, Viale Dell’ Università, 32 I-00185, Roma, Italy; c Department of Mathematics (DISMA), G. L. Lagrange, Politecnico di Torino, Duca Degli Abruzzi 24, I- 10129, Turin, Italy

**Keywords:** Wolf, Parasite dynamics, Free-ranging dogs, Prey community, Drivers of infection, Helminths, Protozoans

## Abstract

Little is known about the impact of infectious diseases on large carnivores. We investigated factors structuring the helminth and protozoan infections of wolves (*Canis lupus*) by using coprological analyses. Faecal samples (n = 342) were analysed from 11 wolf packs belonging to three different geographical and ecological settings in Italy (Abruzzo, Lazio e Molise National Park, PNALM: 4 packs, 88 samples), in France (Mercantour National Park, PNM: 4 packs, 68 samples) and in the U.S.A. (Yellowstone National Park, YNP: 3 packs, 186 samples). Parasites were found in 29.4%–88.6% of the samples and parasite taxa ranged from four to ten in each study area. Taeniidae (*Taenia/Echinococcus*), *Sarcocystis* spp*.* and *Toxascaris leonina* were most common in faecal samples from YNP, whereas *Capillaria* spp., Taeniidae and *Uncinaria stenocephala* were predominant in PNALM. We used generalised linear mixed models to assess the relationship between parasite infection or the number of parasite taxa and selected ecological drivers across study areas. Significant effects illustrated the importance of the ecological factors such as occurrence of free-ranging dogs, diet composition and wolf density, as well as the ancestry of the wolf populations, in shaping parasite-wolf communities. Additional investigations are needed to elucidate the impact of parasitic infections on wolf populations, as well as the role of anthropogenic factors in facilitating parasitic diffusion to apex predators.

## Highlights

• Common drivers affect parasite infection of wolves across different ecological settings.
• Free-ranging dogs, diet composition and wolf density are main drivers of parasite infections.
• Additional factors influence the number of directly/indirectly transmitted parasite taxa across ecological settings.
• *Physaloptera* spp*.* and *Alaria* spp. were so far not described in wolves from PNALM.

## Graphical abstract

**Figure:**
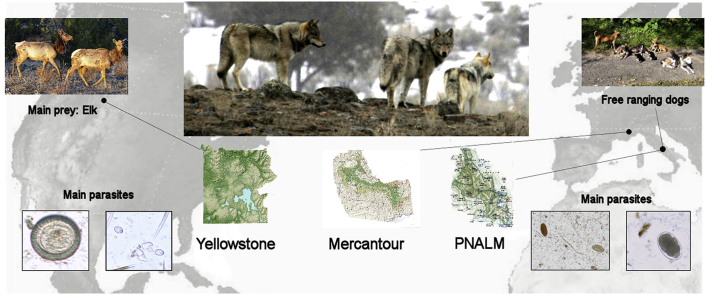

## 1 Introduction

Large carnivores are important to the stability of most ecosystems (Murray et al., 1999) leading to the protection of different endangered populations of wolves, bears and lynxes throughout Europe (Chapron et al., 2014). Environmental and intrinsic correlates of stress, the impact of infectious diseases, anthropogenic mortality, habitat loss and fragmentation are all factors influencing the conservation of these carnivores (Macdonald, 1996; Murray et al., 1999; Molnar et al., 2015). Wolf populations represent three different contexts (Boitani, 2003): (1) existant populations which never went extinct; (2) re-colonizing populations originating from naturally dispersing individuals (Lucchini et al., 2002; Valière et al., 2003; Fabbri et al., 2007; Ciucci et al., 2009); (3) populations originating from translocated animals, following eradication by humans (Bangs and Fritts, 1996). Beyond continental dissimilarities (Craig and Craig, 2005), these different ancestries might have shaped the parasite community in wolf populations (Roberts et al., 2002). Geographical distribution as well as local prey populations are also known to influence the variety of parasites infecting wolf populations (Craig and Craig, 2005; Bryan et al., 2012; Lesniak et al., 2017a).

Parasites infect wolves either directly or indirectly. Direct infections occur via contact with conspecifics, other sympatric hosts or contaminated material (e.g. faeces or vomitus). When parasites are shared by sympatric host species, entire communities might be affected (Holt and Dobson, 2007) and grey wolves (*Canis lupus*) living in sympatry with large, reservoir populations of dogs (*Canis familiaris*) are at a higher risk of infection (Murray et al., 1999; Randall et al., 2004; Cleaveland et al., 2007; Lesniak et al., 2017b). Close physical contact between group members is characteristic of social canids such as wolves and greatly enhances within-pack transmission of pathogens (Johnson et al., 1994). Smell is a crucial sense in wolves, and pack members regularly use urine and faeces to mark their territory. Inspection of faecal markings is frequent along territory edges. The investigation of the anogenital area of conspecifics is part of typical social interactions (Harrington and Asa, 2003). These behavioural characteristics further enhance oro-faecal transmission of potential pathogens (Custer and Pence, 1981b; Kreeger, 2003; Segovia et al., 2003). For example, infection of wolf pups by *Cystoisospora* spp. can lead to hemorrhagic enteritis, diarrhoea, poor growth (Foreyt, 2001) or even death (Mech and Kurtz, 1999).

Indirect infection occurs through feeding on an infected prey, and several helminth parasites are acquired by wolves through feeding on various prey species and scavenging (Segovia et al., 2001; Kreeger, 2003; Craig and Craig, 2005; Moks et al., 2006; Bagrade et al., 2009). While infection by the protozoan *Sarcocystis* spp. is usually considered asymptomatic in canids, infections by several helminth parasites can cause morbidity rather than mortality (Tompkins et al., 2002), exerting a significant impact on energy budgets (Roberts et al., 2002) and hence on population dynamics of canids (Tompkins et al., 2002).

Investigating the association between ecological factors and the infection by pathogens is a basic prerequisite to understand their impact on wolf populations (Murray et al., 1999). A recent comparative survey of the presence of viruses in wolves indicated that density and spatial distribution of susceptible hosts, particularly free-ranging dogs, can be important factors influencing infections in wolves (Molnar et al., 2014). However, the assessment of such drivers through comparison of different geographical regions is complicated by lack of data or by the adoption of different approaches, such as necropsy vs coprology (e.g., Guberti et al., 1993; Schurer et al., 2016; Lesniak et al., 2017a, 2017b). Coprology is a non-invasive technique to assess the occurrence of several pathogens (Torres et al., 2001; Kreeger, 2003; Bryan et al., 2012). It allows large-scale investigations of wildlife populations, otherwise impractical using necropsy, especially where species such as wolves are protected. The faecal samples of carnivores also provide information on infections in prey species (through parasites with an indirect life cycle), or pseudoparasites (parasites of prey) ingested by wolves (Bowman, 2009).

Using coprology, we compared wolf packs belonging to three geographically distinct populations in Italy, France and the USA. Specifically, our aims were: (*i*) to establish a list of parasite taxa hosted by the three wolf populations; (*ii*) to determine the number of parasite taxa and the proportion of faecal samples positive to parasites; (*iii*) to assess drivers (i.e., wolf density, pack size, the presence of free-ranging dogs, wolf population ancestry, and prevailing wolf diet) that may structure parasite communities in wolf populations.

## 2 Materials and methods

### 2.1 Study areas

Eleven wolf packs from three different geographical regions were studied (Table 1): Abruzzo, Lazio e Molise National Park (PNALM) in central Italy, Mercantour National Park (PNM) in south-eastern France and the northern range of Yellowstone National Park (YNP) in north-western United-States. These study areas are located at similar latitudes and show comparable seasonal climatic variations. Pastoralism is important in PNALM and PNM, where livestock is present year-round in some areas. It is inexistent in YNP. During the study period, no significant change in human activities took place within each of the three parks. Red foxes (*Vulpes vulpes*) are present in all three study areas and in YNP additionally coyotes (*Canis latrans*). Free-ranging dogs occur sympatrically with wolves in PNALM and often rely on the same food sources as wolves (P. Ciucci, pers. comm.), but are absent from YNP and very rare in PNM (Table 1).

**Table 1:** Main ecological characteristics of the three national parks where we collected wolf faecal samples to assess the presence of wolf endoparasites: PNALM, central Italy; PNM, France; and YNP, north-western United-States.

National Park	Wolf presence and origin of local population a	Wolf density in winter (ind./1000 km^2^) b	Occurrence of other canids	Occurrence of dogs c	Free-ranging dogs (density/status) d	Wild ungulates e	Livestock	Wolf diet f
PNALM	Always present	50	Fox, dog	Pet dogs, working dogs, stray and feral dogs	High/tolerated, roaming as single or in groups	Chamois, roe deer, red deer, wild boar	Sheep, horses, cattle, few goats	Varied: mainly wild boar, followed by red deer, roe deer, and livestock
PNM	Natural recolonisation from the Apennines (Italy) since 1992	10.5	Fox, dog	Pet dogs and working dogs	Very low/prohibited (controlled)	Chamois, European mouflon, roe deer, red deer, wild boar, ibex	Sheep, few goats and cattle	Varied: mainly chamois and roe deer, but also red deer, ibex, European mouflon, wild boar, and few sheep and goats
YNP	Reintroduction from the Canadian Rockies in 1995–1996	50	Fox, coyote, dog	Pet dogs	Inexistent/prohibited (controlled)	Elk, bison, mule deer, white-tailed deer, moose, pronghorn antelope, bighorn sheep, mountain goat	None	Specific: ≥ 96% elk, few bison, mule deer, white-tailed deer, and moose

a PNALM: Boitani (2003); PNM: Houard and Lequette (1993); YNP: Berger and Smith (2005).

b PNALM: mean estimated value (Grottoli, 2011); MNP: mean number of wolves per pack divided by the mean estimated size of packs' territory (260–350 km^2^; ONCFS Réseau Loup/Lynx, 2007; Duchamp et al., 2012); YNP: mean value for the northern park range (Coulson et al., 2011).

c Dogs travelling with tourists are prohibited in PNALM, allowed in the buffer zone but excluded from the core area in PNM, and restricted to a range of 100 yards off roads and parking lots in YNP. Working dogs are shepherd dogs and livestock-guarding dogs.

d PNALM: Boitani et al. (1995); Boitani et al. (2006); PNM: G. Millischer pers. comm.; YNP: Bangs et al. (2005).

e Chamois (*Rupicapra rupicapra*), roe deer (*Capreolus capreolus*), red deer (*Cervus elaphus*), wild boar (*Sus scrofa*), European mouflon (*Ovis orientalis*), alpine ibex (*Capra ibex*), elk (*Cervus canadensis*), bison (*Bison bison*), mule deer (*Odocoileus hemionus*), white-tailed deer (*Odocoileus virginianus*), moose (*Alces alces*), pronghorn antelope (*Antilocapra americana*), bighorn sheep (*Ovis canadensis*), and mountain goat (*Oreamnos americanus*).

f Wolf faecal samples collected in PNALM and PNM were submitted to dietary analyses (PNALM: P. Ciucci, unpublished data; PNM: ONCFS Réseau Loup/Lynx, 2004; 2006). In YNP, main prey species were assessed through close observation of local wolf packs (Smith et al., 2008, 2009, 2010).

### 2.2 Investigated wolf populations and packs

The three protected wolf populations differ by their origin (Table 1). Wolves never disappeared from PNALM (Zimen and Boitani, 1975) and acted as a source for the natural recolonisation of the northern Apennines and the Alpine range, including PNM (Fabbri et al., 2007; Ciucci et al., 2009). In YNP, wolves were reintroduced in 1995 and 1996 through the release of de-wormed individuals (D. Smith, U.S. National Park Service, pers. comm.) captured in Alberta and British Columbia, Canada (Bangs and Fritts, 1996). The density of wolves was similar in PNALM and YNP, while it was almost five times lower in PNM (Table 1). For the scope of this study, we defined one wolf pack as a minimum of one male and one female travelling together. Based on the quality and quantity of faecal samples we obtained, we selected four packs in each of the two European national parks (PNALM: Iorio, Orsara, Villavalelonga, and Mainarde packs; PNM: Haute Tinée, Moyenne Tinée, Vésubie-Roya, and Vésubie-Tinée packs). These packs comprised a minimum of 25 wolves in PNALM and 18–23 in PNM. On the northern range of YNP, we studied three different packs (Slough Creek, Druid Peak, Blacktail Deer Plateau) comprising 36–39 wolves. (Supplementary data Table 1). To locate the packs, we relied on previous knowledge by local wolf researchers and used snow-tracking, howls and bird activity near wolf kill-sites, or accompanied local field crews who used telemetry. In the YNP, the packs were observed daily, from dawn to dusk, whenever weather conditions and distance to the animals (i.e., 100–1500 m) allowed it (Baan et al., 2014). Since sample collection started in early winter (see below), all samples were from individuals of over six months of age.

### 2.3 Faecal sampling

For PNM and PNALM, we used wolf faecal samples collected by scientists and rangers for different projects during winter (i.e., October 2006–March 2007; Ciucci and Boitani, 2009; Grottoli, 2011; Duchamp et al., 2012). In YNP, we collected samples during winter (December–March) 2007–2008 and winter 2008–2009 (Supplementary data Table 1). In PNALM and PNM, we collected most samples within 24–48 h following snowfalls while snow-tracking the packs, thus directly identifying the contributing pack (Ciucci and Boitani, 2009; Duchamp et al., 2012). In the absence of snow cover, we collected samples at known scent posts, at wolf kill- or scavenging-sites or during opportunistic surveys along pathways (Grottoli, 2011; Duchamp et al., 2012). In YNP, we collected faecal samples within hours following direct observation and filming of contributing individuals. We avoided sample collection when wolves not belonging to the studied packs were known to have used the area. We considered only well-preserved faecal samples at the time of collection and discarded those partly consumed by birds, dried out, or exposed to rain or temperatures above freezing. Scats composed mostly of hair (estimated as > 90% of the scat volume) or lying less than 50 cm away from one another (to avoid potential cross-contamination of samples) were excluded. The handling of samples was carried out wearing thick protection gloves and breathing mask. All samples were stored on the day of collection at −20 °C in labelled plastic bags and were kept frozen until analysis.

### 2.4 Coproscopy

We prepared the faecal samples for coproscopy in a biological safety hood Class II. Of each homogenised faecal sample, we used 1.55 ± 0.05 g and for the parasite concentration, a modified sodium acetate - acetic acid - formaldehyde (SAF) technique (Yang and Scholten, 1977). We prepared a stained and an unstained preparation of each sample (Truant et al., 1981). We mixed a drop of the concentrated faecal solution with a drop of physiological saline solution for the unstained preparations and a drop of Lugol solution (dilution 1:5) (Ash and Orihel, 1991) for the stained preparations. We systematically scanned the coverslipped (18 × 18 mm) preparations at ×100 magnification, using a calibrated Olympus BX50 microscope, and confirmed each observation at 400x magnification. We identified helminth eggs based on their size, colour, shape, the aspect of their content and the structure of the shell surface. We classified eggs from the Taeniidae family together, as they cannot be differentiated by microscopic examination (Foreyt, 2001; Kahn and Line, 2005; Bowman, 2009). All other helminth eggs and protozoan cysts we identified to genus or species level (Leger et al., 1977; Garcia and Ash, 1979; Thienpont et al., 1979; Bailenger, 1982; Ewing, 1986; Campbell, 1991; Uga et al., 2000; Foreyt, 2001; Bowman, 2000a, 2000b; 2009; Traversa et al., 2010).

We determined the number of parasite taxa from a given study area. The proportion of samples with at least one egg or cyst of a parasite taxon we described as N+/N, with N+ being the number of positive faecal samples and N the total number of analysed samples. For each value, we calculated 95% confidence intervals (Motulsky, 1995), following a binomial distribution for large sample size. For further details on coprological analysis, see Molnar (2012) and Molnar et al. (2014, 2015).

### 2.5 Statistical analyses

We developed models to assess the relationship between measures of occurrence and abundance of parasite taxa in wolf scats (response variables) and the most plausible dominant ecological factors in each wolf population (Table 2). We considered as response variables: *i*) infection status, coded as 0 for no parasite detected vs 1 for one or more parasite taxa detected; *ii*) parasite taxa (i.e., the number of identified taxa in a faecal sample). We used as explanatory variables free-ranging dogs (presence vs absence), diet composition (varied vs specific; Table 1), wolf density, pack size, and Park ID; the latter was meant as a proxy of wolf population ancestry, even though it might account for other factors that differ across study sites and that we did not consider. To assess the relationship between infection status (i.e., a binary response variable) and the exploratory variables we used generalised linear mixed-effect models (GLMM) with a logit link, whereas to test for the above effects on parasite taxa (i.e., a Poisson-distributed response variable) we used a GLMM with a log link. To account for pseudoreplication and the nested nature of our data, we included a random intercept for pack ID, nested within the study area, in our GLMMs. All models we developed using the *lme4* package (Bates et al., 2015) in R (R Core Team, 2018).

**Table 2:** Models developed to investigate the relationship between the occurrence of parasites detected in wolf scats and ecological factors using datasets of three different wolf populations: PNALM (winter 2006–2007); PNM (winter 2006–2007); YNP (winters 2007–2008 and 2008–2009).

Classes of models	Response variable	Explanatory variables a
1. Overall ecological effects	Infection status b	Pack ID*, wolf density, free-ranging dogs, park ID, diet
	Parasite taxa c	
2. Ecological effects separately for *directly* vs *indirectly* transmitted parasites	Infection status b	Same as for the models of class 1
	Parasite taxa c	
3. Ecological effects separately for selected groups of parasites	Infection status b	Same as for models of class 1
	Parasite taxa c	
		

^a^ Random factors in GLMM (Generalised Linear Mixed Models) are marked with *.

b Code 0 (no parasites detected in a faecal sample) vs 1 (≥1 parasite taxa detected).

c The total number of parasite taxa identified in a faecal sample.

Using these models, we also investigated the same relationships separately for directly vs indirectly transmitted parasites. Also, as Taeniidae and*, Sarcocystis* spp. were found in all three study areas, we also developed the above-described models for each of these taxa separately (Table 2).

For each group of models, we tested models composed of all single effects and two covariates interactions among those deemed most plausible and all possible subsets. We then selected the most parsimonious models using the Akaike Information Criterion corrected for small sample size (AICc) and averaged models with lowest AICc value (i.e., ΔAICc < 2; Burnham and Anderson, 2002). Averaged coefficients in the final models were deemed significant if their 95% confidence interval (CI) did not include zero. We used the Nagelkerke Pseudo-R^2^ (Nagelkerke, 1991) to assess how each of the averaged models fit the data. We also checked for overdispersion of the fitted models using the sum of squared Pearson residuals which, under the hypothesis of no overdispersion, is distributed as a chi-squared with df equal to the residuals’ df minus one. For all models, the test failed to reject the null hypothesis (0.41 ≤ p ≤ 1.0), with ratios of the sum of squared over the df, that should be 1 under the null, ranging 0.63–1.01.

Finally, we used the Chi-square test to assess difference in the number of positive samples between study areas. We caution that our results pertain to the sampled scats but, due to their potential lack of independence among each other, they are not necessarily representative of the whole population.

### 2.6 Ethics statement

The collection of faecal samples is a non-invasive procedure and did not require approval by animal ethics committees. The wolf is protected in all three study areas. In PNALM research was approved by the national park authority (Determination no. 38 of 24 March 2003). No specific permission was required for the collection of faecal samples in PNM. In YNP, in agreement with the park's policy; permits YELL-2007-SCI 5716, YELL- 2008-SCI 5716, and YELL-2009-SCI 5716 were delivered by the authority of the national park.

## 3 Results

### 3.1 Parasite taxa and proportion of parasite-infected faecal samples

**Fig. 1:**
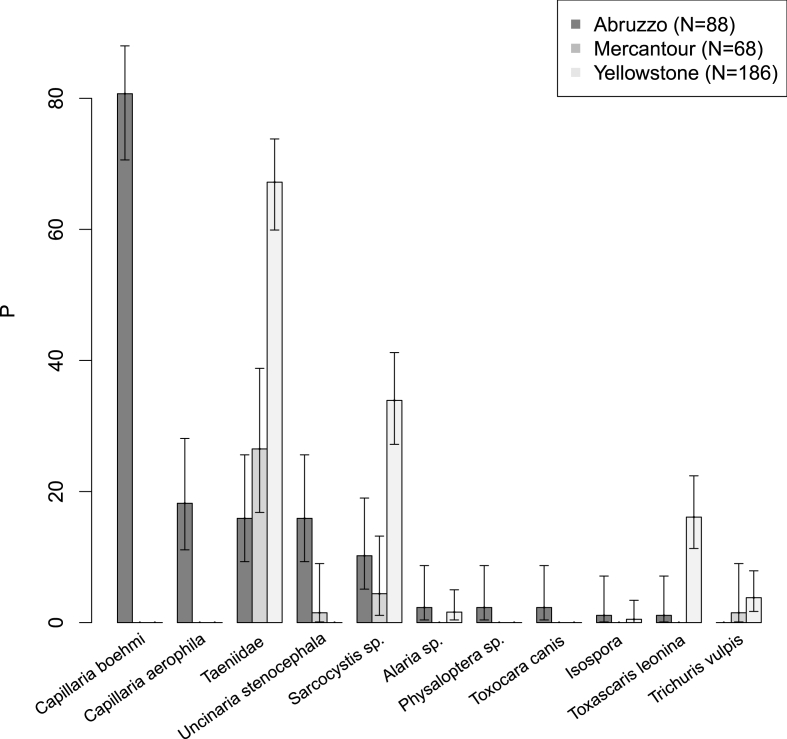
Canid endoparasites detected in faecal samples of three different wolf populations from PNALM (2006–2007), PNM (2006–2007), and YNP (2007–2009). The total number of analysed samples (N), the proportion (P) and corresponding 95% confidence intervals (CI) are specified. P and CI are expressed as percentages (%).

In 342 analysed wolf scats (PNALM: N = 88; PNM: N = 68; YNP: N = 186), we identified 11 different parasite taxa, from four in PNM to ten in PNALM (Fig. 1; Supplementary data Table 2). The proportion of positive samples did not differ (χ^2^ = 1.905, df = 1, p = 0.167) between the PNALM and YNP, that shared the highest values (88.6% and 81.7%, respectively), whereas the proportion in PNM (29.4%) was lower compared to the two other national parks (55.098 ≤χ^2^ ≤ 58.337 1 ≤ df ≤ 1, 0.000 ≤ p ≤ 0.000). At the pack level, we detected on average 3.8 (±2.6 SD) parasite taxa per pack, ranging from 1 in the Vésubie-Roya and Moyenne-Roya packs (PNM) to 10 in the Orsara pack (PNALM); accordingly, proportion of positive samples ranged from 14.3% in the Vésubie-Roya pack (PNM) to 100% in the Orsara pack (PNALM). Taeniidae (*Taenia/Echinococcus* spp.), *Sarcocystis* spp*.* and *Toxascaris leonina* were most common in faecal samples from YNP, whereas *Capillaria* spp. and *Uncinaria stenocephala* were more common in PNALM. The two *Capillaria* species, *Physaloptera* spp. and *Toxocara canis* were only found in PNALM. *Trichuris vulpis* was only found in YNP. All parasites found in PNM were also detected in PNALM. (Fig. 1; Supplementary data Table 2.).

The trematode *Dicrocoelium dendriticum* was the most commonly detected pseudoparasite in the European national parks. In YNP, Capillariidae were the only pseudoparasites detected. The highest diversity of pseudoparasites was found in PNALM (Table 3). At the pack level, we detected on average one to two pseudoparasite taxa per pack, ranging from none in the Slough Creek pack (YNP) to 5 in the Mainarde pack (PNALM).

**Table 3:** Pseudoparasite helminths identified in 342 wolves’ faecal samples of three different wolf populations from PNALM (2006–2007), PNM (2006–2007), and YNP (2007–2009). The total number of tested samples (N), the number of samples that tested positive (N+), proportion (P) and confidence intervals (CI) are figured. P and CI are expressed as percentages (%).

Pseudoparasites taxa		PNALM (N = 88)			PNM (N = 68)			YNP (N = 186)			Total (N = 342)		
		N+	P	CI	N+	P	CI	N+	P	CI	N+	P	CI
Trematoda	*Dicrocoelium dendriticum*	14	15.9	9.3–25.6	11	16.2	8.7–27.5	0	0	–	25	7.3	4.9–10.7
Nematoda	Capillariidae a	1	1.1	0.1–7.1	1	1.5	0.1–9.0	3	1.6	0.4–5.0	5	1.5	0.5–3.6
	*Metastrongylus* spp.	4	4.5	1.5–11.9	0	0	–	0	0	–	4	1.2	0.4–3.2
	*Nematodirus* sp*.*	0	0	–	1	1.5	0.1–9.0	0	0	–	1	0.3	0.0–1.9
	*Toxocara cati*	1	1.1	0.1–7.1	0	0	–	0	0	–	1	0.3	0.0–1.9
	*Trichuris suis*	3	3.4	0.9–10.3	0	0	–	0	0	–	3	0.9	0.2–2.8
Pseudoparasite taxa		5			3			1			6		

a Eggs identifiable to the family level only, but that do not correspond to any genus known to infect canids.

### 3.2 Drivers of parasite infection status and parasite taxa

Chances of parasite infection were higher in areas where free-ranging dogs occurred and also where wolf populations were thriving on a specialised rather than a more diversified diet. (Table 4a), but we failed to reveal pack-size and wolf density effects. However, the number of parasite taxa detected in wolf faeces was cumulatively affected by wolf density, occurrence of free-ranging dogs, and diet composition (Table 4b). Wolves living in sympatry with free-ranging dogs were infected by more parasite taxa, and wolves living at higher densities also tended to be infected by a higher number of parasite taxa. Finally, wolves consuming a more diversified array of prey species hosted a lower number of parasite taxa compared to wolf populations predating on one or two ungulate species (Table 4b).

**Table 4:** Model selection and drivers of parasite infection status detected in faecal samples from three wolf populations in PNALM (2006–2007), PNM (2006–2007), and YNP (2007–2009). All models (see Table 2 for all tested covariates) were fitted with a random intercept for pack identity nested within the study area. Only candidate models with ΔAICc <10 are shown. We used candidate models with ΔAICc ≤2 for model coefficient averaging. R^2^: Nagelkerke Pseudo-R^2^ (selected models only); K: number of estimable parameters; AICc: Akaike information criterion adjusted for small sample sizes; ΔAICc = (AICc) – (AICc)min; w: Akaike weight.

a) Effects on infection status for all parasites.						
Fixed-effects parameters	R^2^	K	log-likelihood	AICc	ΔAICc	w
Free-ranging dogs + Diet	0.258	3	−155.7136	321.427	–	1

Fixed-effect parameters	β	SE	95% confidence interval	
			lower	upper
Intercept	1.498	0.190	1.126	1.869
Free-ranging dogs a	3.005	0.441	2.141	3.869
Diet b	−2.448	0.343	−3.120	−1.777

b) Effects on parasite taxa for all parasites.						
Fixed-effects parameters	R^2^	K	log-likelihood	AICc	ΔAICc	w
Wolf Density	0.233	2	−404.050	816.100	–	0.598
Free-ranging dogs + Diet	0.230	3	−403.447	816.894	0.794	0.401

Fixed-effect parameters	Β	SE	95% confidence interval	
			lower	upper
(intercept)	−0.950	0.969	−2.85	0.949
Wolf density	0.040	0.007	0.026	0.054
Free-ranging dog a	1.628	0.275	1.090	2.167
Diet b	−1.437	0.274	−1.974	−0.899

c) Effects on parasite taxa for directly transmitted parasites						
Fixed-effects parameters	R^2^	K	log-likelihood	AICc	ΔAICc	w
Free-ranging dogs + Pack size	0.727	3	−193.783	397.565	0	0.698
Pack size + Park	0.703	4	−193.622	399.244	1.678	0.302

Fixed-effect parameters	β	SE	95% confidence interval	
			lower	upper
(intercept)	−4.299	2.119	−8.452	−0.146
Free-ranging dogs a	4.348	0.717	2.942	5.754
Pack size	0.259	0.081	0.100	0.419
YNP c	−4.732	1.010	−6.712	−2.752
PNM c	−3.850	1.020	−5.849	−1.850

d) Effects on parasite taxa for indirectly transmitted parasites						
Fixed-effects parameters	R^2^	K	log-likelihood	AICc	ΔAICc	w
Diet	0.274	2	−341.839	691.678	0	0.685
Park	0.275	3	−341.615	693.230	1.552	0.315

Fixed-effect parameters	β	SE	95% confidence interval	
			lower	upper
(intercept)	−0.213	0.588	−1.366	0.940
Diet b	−1.326	0.161	−1.642	−1.010
PNM c	−0.202	0.305	−0.799	0.396
YNP c	1.249	0.195	0.866	1.631

a Reference: Free-ranging dogs absent.

b Reference: Specific diet.

c Reference: PNALM (Italy).

The number of directly transmitted parasite taxa per faecal sample was again cumulatively affected by the occurrence of free-ranging dogs in the area, but also by pack size and Park ID (Table 4c). Specifically, the number of parasite taxa was much higher in areas with sympatric free-ranging dogs and tended to increase with pack size; in addition, a study area effect revealed that in the PNALM, independently from the occurrence of free-ranging dogs, other factors contributed as well to the highest number of directly transmitted parasite taxa we revealed, compared to PNM and YNP (Table 4c). The number of indirectly transmitted parasite taxa was affected by the type of diet and the Park ID. Wolves thriving on a more diversified diet tended to have a lower number of indirectly transmitted parasites taxa compared to wolves living on one or a few prey species; in addition, wolves in YNP hosted a higher number of indirectly transmitted parasite taxa compared to PNALM (Table 4d).

### 3.3 Drivers of infection for selected groups of parasites

The infection status concerning *U*. *stenocephala* was only affected by the occurrence of free-ranging dogs. Wolves living in areas without free-ranging dogs had much lower chances of being infected (Supplementary data Table 3). Similarly, infection status for *T. leonina* was solely affected by pack size, with larger packs having a higher chance of being infected (Supplementary data Table 4).

## 4 Discussion

To our knowledge, this is the first broad investigation of helminth and protozoan parasites in grey wolves of three different geographical regions. The data complement and broadens several other geographically more restricted studies using necropsy or coprology (Guberti et al., 1993; Stronen et al., 2011; Bryan et al., 2012; Schurer et al., 2016; Lesniak et al., 2017a, 2017 b; Al-Sabi et al., 2018). Our findings correlate well with the ancestry and the ecology of the three wolf populations. The PNALM population is the oldest one with free-ranging dogs widely present, followed by YNP and PNM populations. Significant differences were detected in the overall number of parasite taxa and the extent of their infection across the three geographic areas we considered.

### 4.1 Factors shaping parasite communities

Our models demonstrated common drivers across all parks. The presence of sympatric free-ranging dogs, as well as reliance by wolves on one or two main prey species, compared to a more diversified diet, positively correlated with both infection status and the number of parasite taxa we detected in faecal samples. Wolf density and park may also play a role, however on a more limited scale.

#### 4.1.1 Free-ranging dogs

The impact of free-ranging dogs is illustrated by the surprisingly high proportion of samples with *C. boehmi* (80.7%), exclusively detected in PNALM. Only recently, Al-Sabi et al. (2018) reported a high proportion of 60% of *C. boehmi* in wolves of Sweden. The high percentage of this mainly directly transmitted parasite suggests a very efficient transmission and an important contamination of the environment. *C. aerophila* was always present, at a lower proportion, as a coinfection in samples with *C. boehmi*. *C. aerophila* was reported in wolves in Eastern Europe (Shimalov and Shimalov, 2000; Popiołek et al., 2007; Bagrade et al., 2009; Szafrańska et al., 2010), Russia and North America (Peterson et al., 1998; Craig and Craig, 2005). As the investigation of faeces is commonly used to convey information in canids, these parasites can easily be acquired through inhalation of infective eggs from deposited scats. During the study period, *Capillaria* species were not common parasites of foxes in central and Northern Italy (Di Cerbo et al., 2008; Magi et al., 2009), even though they have been more recently reported (Veronesi et al., 2014; Magi et al., 2015). The consistent population of free-ranging dogs in Italy (Verardi et al., 2006; Corrain et al., 2007), including PNALM (Boitani et al., 1995), served probably as a reservoir for *C. boehmi*. In 2013 the death of several wolves in PNALM was attributed to a CDV outbreak (Di Sabatino et al., 2014). *Capillaria* spp. infections might have been a co-factor. The knowledge of the range of hosts and the geographic distribution of *C. aerophila* is still incomplete.

#### 4.1.2 Diet

The importance of the diet on the infection status and the number of parasite taxa was confirmed through the dominant proportion of the Taeniidae and *Sarcocystis* spp. detected in all three investigated areas. These two taxa, with an indirect life cycle, are known to infect a broad array of ungulate and carnivore species worldwide. The genera *Taenia* and *Echinococcus* are widespread intestinal parasites of canids and have frequently been reported in wolves, (Foreyt, 2001; Kahn and Line, 2005; Bowman, 2009). Both genera include different species described in wolves in Europe and North America (Guberti et al., 1993; Custer and Pence, 1981b; Lesniak et al., 2017a).*Taenia* spp. require an herbivore or omnivore intermediate host, such as ungulates, lagomorphs, and rodents (Guberti et al., 1993; Marquard-Petersen, 1997; Craig and Craig, 2005). The high proportions of positive samples we detected in YNP, two to three times higher than in PNALM and four times higher than in PNM, is closely linked to the selection of elk as a primary prey (>96%) by wolves on the northern range of YNP (Smith et al., 2008, 2009, 2010). It also suggests a high level of infection of the YNP elk population. In line with the lower proportions of infected samples also reported for Canada (Stronen et al., 2011; Bryan et al., 2012), lower infection extent in PNALM and PNM are in agreement with the more diversified wolf diets in these regions, some of them not infected by Taenids.

*Echinococcus granulosus* is widespread in Italy and in particular in the Abruzzo region, where it is closely associated to sheep and cattle grazing and numerous sheepdogs (Guberti et al., 1992; Garippa et al., 2004; Garippa and Manfredi, 2009). This parasite has also been detected in wolves in PNALM (L. Gentile, PNALM Veterinary Service, pers. comm.). In PNM no information is available, but in YNP *E. granulosus* was detected in some wolves (https://www.nps.gov/yell/learn/ys-24-1-infectious-diseases-of-wolves-in-yellowstone.htm). Although *Echinococcus* spp are important zoonotic agents causing human echinococcosis, to our knowledge, no cases of human infection have been recently reported from these parks.

The presence of *Sarcocystis* spp. in all three study areas was not surprising. Numerous species of *Sarcocystis* infect a wide range of prey species around the world, including domestic and free-ranging ungulates (Gajadhar et al., 2015). Infection of wolves by *Sarcocystis* spp. has been reported in different geographical regions (Emnett, 1986; Kreeger, 2003; Stronen et al., 2011 Bryan et al., 2012; Schurer et al., 2016; Lesniak et al., 2017a, 2017 b) and are also well-known parasites of dogs, foxes and coyotes (Fayer and Johnson, 1975; Ewing, 1986; Rajković-Janje et al., 2004). The proportion of samples with *Sarcocystis* spp. of YNP are similar to the values reported for Canada (36.5–43.7%; Stronen et al., 2011; Bryan et al., 2012). Lesniak et al. (2017a) found 95% of necropsied wolves found dead in Germany being positive for *Sarcocystis* spp., where red deer and fallow deer (*Dama dama*) are the main prey (Lesniak et al., 2017a). Patency of *Sarcocystis* is of a few days (Emnett, 1986). The time of the collection of the faecal samples has a direct impact on the parasite detection, besides well-known methodological differences (see point 4 below). The more diversified diet of wolves in PNALM and PNM, relative to YNP, could have contributed to the lower proportion of *Sarcocystis* spp. Modelling the numbers of parasites with an indirect life cycle (mainly Taeniidae and *Sarcocystis*), indicated that wolves with a more diversified diet hosted a lower number of parasite taxa. Indeed, wolves in YNP, perhaps due to their more closely associated predator-prey relationship, showed a higher probability of being infected by more than one parasite taxon with an indirect life cycle.

The proportion of positive samples and the number of pseudoparasite taxa were highest in PNALM, followed by PNM and lower in YNP, mirroring an increasing gradient in diet specificity across the regions. Although infection of wolves through coprophagy cannot be excluded, the detection of pseudoparasite eggs infecting suids (swine and wild boar), such as *Trichuris suis* and *Metastrongylus* spp. (Table 3), provides direct information on the diet of wolves. Detection of more generalist parasites such as *D. dendriticum*, *Nematodirus* spp. or Capillariidae, that infect a broad range of hosts, is an indicator of their presence in potential wolf prey species in the area.

#### 4.1.3 Wolf density

An elevated host density usually facilitates the spread of parasites in a population (Tompkins et al., 2002) and the transmission of new parasite species (Roberts et al., 2002). A significant correlation between cortisol metabolite levels (indicators of stress) and a higher number of parasite taxa (two or more) in faecal samples of PNALM was already described (Molnar et al., 2015).

In addition to wolf density at the population level, a higher number of members within a pack corresponds to higher chances of infection by one or more parasites taxa. Taxonomically related host species are susceptible to be infected by the same parasite species (Freeland, 1983), and part of the parasite fauna of wolves is also found in other canids such as dogs, red foxes and coyotes (*Canis latrans*) (Erickson, 1944; Custer and Pence, 1981b; Campbell, 1991; Guberti and Poglayen, 1991; Guberti et al., 1993; Di Cerbo et al., 2008; Bryan et al., 2012). Our findings indicate that the presence of free-ranging dogs was a relevant driver of both infection status and the number of parasite taxa detected in wolf faeces. Contamination of the environment with directly transmitted parasites is enhanced by sympatric populations of canids and thus facilitates infection of wolves by shared parasites. In all three investigated areas, wild canids may importantly contribute to this environmental effect. In central Italy, free-ranging dogs most likely use similar prey species as wolves (Ciucci and Boitani, 1998), and in PNALM both free-ranging dogs and wolves scavenge on abandoned livestock carcasses (P. Ciucci, pers. comm.), enhancing the impact of sympatric canids on infection of wolves.

#### 4.1.4 Park

The differences in parasite numbers between the three investigated areas indicated that additional different geographical and biological factors structure the parasite community of wolves. Ancestry of the studied populations, other ecological correlates, presence and diversity of sympatric susceptible canids are factors that may explain these differences. Our models on all parasite taxa (i.e., both directly and indirectly transmitted ones) revealed no park effect on infection status, nor on the number of parasite taxa detected in faecal samples. However, by separating directly and indirectly transmitted parasites, we did reveal a park effect, and different factors affected the number of parasite taxa in the studied wolf populations. Wolves living in PNALM had higher chances of hosting a higher number of directly transmitted parasites (mainly *Capillaria* spp. and *U. stenocephala*). Wolves in YNP had higher chances of hosting indirectly transmitted parasites (mainly Taeniidae and *Sarcocystis*). Guberti et al. (1993) suggested that long-established wolf populations might harbour helminths different from newly settled populations. As the wolf never disappeared from central Italy (Boitani, 2003), the high number of parasite taxa that we reported in PNALM might reflect a long-term co-evolution between the wolf and its parasite community. Equally high parasite numbers were reported in wolves elsewhere in Europe (Segovia et al., 2001, 2003; Moks et al., 2006; Bagrade et al., 2009) and North America (Stronen et al., 2011; Bryan et al., 2012), in all cases associated with long-established wolf populations. In YNP, the parasite community of wolves grew from zero, as reintroduced wolves were de-wormed before their release in the park in 1995 and 1996 (D. Smith, pers. comm.). It is therefore not surprising that the number of parasite taxa we detected in YNP was lower compared to long-established, untreated, populations in Canada (Stronen et al., 2011; Bryan et al., 2012). In the absence of conspecifics, Yellowstone wolves likely acquired parasites maintained in the environment by coyotes, their closest relatives in the area. Indeed, all parasites detected in the YNP packs are known coyote parasites (Erickson, 1944; Thornton et al., 1974; Arther and Post, 1977; Hudkins and Kistner, 1977; Conder and Loveless, 1978; Dubey, 1980; Custer and Pence, 1981a; Radomski and Pence, 1993), and some of them also infect foxes in North America (Erickson, 1944). Wolves dispersing into YNP (Jimenez et al., 2017) might have introduced into local packs some of the detected parasites, such as the helminth *T. vulpis*, a less common parasite of coyotes (Custer and Pence, 1981a). The total proportion of infected samples in YNP (81.7%) was coherent with previous findings in wolves from North America (91.0% by necropsy, Rausch and Williamson, 1959; 95.0%, Custer and Pence, 1981b). This proportion was higher than the one reported for British Columbia (62.6%, Bryan et al., 2012) with the predominance of the same two parasite taxa: *Sarcocystis* spp. and Taeniidae. Our results show that Taeniidae and *Sarcocystis* spp. were especially prevalent in the elk population of YNP.

The lowest number of parasite taxa (only four) and the lowest proportion of infected samples were found in PNM. Reinfection of wolves is helped by their fidelity to den sites and rendezvous areas in well-established packs (Custer and Pence, 1981b; Kreeger, 2003; Segovia et al., 2003). During the recent wolf re-colonisation process of the French Alpe, such fidelity might not have been immediately established, and less common parasites may have consequently failed to survive in the environment. Small populations of hosts that recolonise new areas usually harbour a subset of the total variety of parasites present in the source population (Roberts et al., 2002). If the colonised area is suitable for the parasite life cycle, most prevalent species are likely transferred from the source population, whereas less common ones are expected to disappear in the recolonising population (Roberts et al., 2002). A dispersal corridor connects the wolf population of PNM with the one of the Apennines in Italy, from where dispersers founded the packs in PNM through a natural re-colonisation process (Ciucci et al., 2009). The absence of *Capillaria* spp. in PNM might be due to lack of infection in dispersing wolves during the re-colonisation period. Heavily infected wolves may have been physically less efficient in dispersing to PNM. Both *Capillaria* species infect the respiratory system of wolves and can severely impair infected hosts (Bowman, 2000a, 2009). The overall lower wolf density and the lack of free-ranging dogs in PNM both prevent the contamination of the environment by these parasites. Since the presence of *Capillaria* spp. in foxes has not been reported from PNM, their presence should be further evaluated.

### 4.2 Individual parasites

*Cystoisospora* spp. was, beside *Sarcocystis* spp., the only other protozoan parasite detected in one sample each in PNALM and YNP. This protozoan was identified in Canada (Bryan et al., 2012) and in the United States, likely causing the death of wolf pups (Mech and Kurtz, 1999). Hermosilla et al. (2017) reported *Cystoisospora* spp. also in.

Croatian wolves. Consistent with our results, *T. leonina* and *U. stenocephala* were previously reported in wolves from Italy and Germany (Guberti et al., 1993; Lesniak et al., 2017a). *T. leonina* was also detected in studies in Canada (Stronen et al., 2011; Bryan et al., 2012). Reports of *T. canis* in wolves vary markedly from 0.3% to 33% (Stronen et al., 2011; Bryan et al., 2012; Guberti et al., 1993; Lesniak et al., 2017a; Paoletti et al., 2017), and this parasite was detected in over half of necropsied foxes from Northern Italy (Di Cerbo et al., 2008). The absence of *T. canis* in PNM and YNP and the small proportion of positive samples in PNALM suggest either a low prevalence of this parasite in the studied wolf populations or a possible artefact due to the timing of our sampling. Infection of *T. canis*, more common in younger canids compared to adults (Guberti et al., 1993; Bowman, 2009), may be lethal to pups (Foreyt, 2001; Kreeger, 2003; Bowman, 2009). In winter, infected pups may either succumb to infection or overcome it, although we know that no wolf pup died in the studied packs from YNP. *Physaloptera* spp. and *Alaria* spp. found in wolf samples from PNALM and YNP were not reported for Italy before. *Physaloptera* spp. were reported for Persia, Northern Asia (Kreeger, 2003), Greece and North America (Erickson, 1944; Schurer et al., 2016), while *Alaria* spp. have been described in wolves worldwide and also in Manitoba, Canada (Stronen et al., 2011). *T. vulpis* was not found in PNALM and in only one sample in PNM. This might indicate that the helminth was not widespread, since the sensitivity of coprology for whipworms is relatively high (43 ± 3%; Wilson et al., 2002).

### 4.3 Unidentified nematode larvae

The identification of unidentified nematode larvae would have needed a thorough morphological or molecular examination. They could be first-stage larvae of hookworm (in our samples: *U. stenocephala*) that hatched from eggs in faeces. Free-living or plant nematodes could have been migrating into the scat from the environment (Traversa et al., 2010), larvae stemming from infested prey, or parasites of wolves eliminated as larvae. Different parasites of the dog are present as larvae in faeces, including *Oslerus osleri, Strongyloides stercoralis, Angiostrongylus vasorum* or *Crenosoma vulpis* (Traversa et al., 2010) and most of these parasites have been reported in wolves (Erickson, 1944; Shimalov and Shimalov, 2000; Segovia et al., 2001; Popiołek et al., 2007; Bagrade et al., 2009). Sampling in winter most likely prevented the development of nematode larvae and the contamination of collected faecal (Marquard-Petersen, 1997). If the detected unidentified larvae were considered as wolf parasites, the reported helminth proportion would double in PNM, but remain about the same in PNALM and YNP.

### 4.4 Methodological caveats of coprology

Torres et al. (2001) showed in wild canids that coprological prevalences were significantly lower than those found by necropsy, but they conclude that in wild canids coprological surveys provide an acceptable approximation to the real parasite fauna. Additionally, the number of parasite taxa is underestimated since several parasites could only be determined to the family (Taeniidae) or genera (e.g. Sarcocystis) level. Therefore, our results represent an underestimation of the real extent of parasite presence. In Italy, *M. lineatus, D. caninum,* and *Ancylostoma caninum* were detected in less than 16% of analysed guts of wolves (Guberti et al., 1993). Magi et al. (2009) reported an elevated proportion of *M. lineatus* and *D. caninum* in red foxes through necropsy (45.4 and 57.3%) but found no eggs in faecal samples of the same individuals. The low sensitivity of coprology probably explains their absence in our data. Eggs and larvae of *A. caninum* are destroyed by freezing (Bowman, 2009) explaining their absence from our samples, collected in winter and stored frozen.

The term prevalence is sometimes erroneously used in the literature to describe the proportion of faecal samples containing parasites. We avoided the use of this term because the collected faecal samples are not necessarily an unbiased and representative sample of the entire wolf population. Nevertheless, we believe they provide a practical yet useful assessment of the extent of infection within a host population, especially if consistently used to compare different host populations.

## 5 Conclusions

Even if the actual presence of parasites may be underestimated in our study due to the low sensitivity of coprology, our findings indicate high levels of infection by a high number of parasite taxa in PNALM and YNP. Besides geographical, historical and wolf population parameters, the presence of free-ranging dogs and the diversity of prey species influence the parasite fauna of wolves. To better assess the epidemiological factors shaping the parasitic fauna and their impact on wolves, investigations of infections in sympatric canid populations (free-ranging dogs, coyotes, and foxes) and their preferred prey should be undertaken in all three study areas.

## Funding

This work was supported by the laboratories of animal physiology, parasitology and eco-ethology of the University of Neuchâtel, by the Fondation Gérard Pierre, the Société académique neuchâteloise and private donors.

## Conflicts of interest

Declarations of interest: none.
