# Supplementary material for: Correlates of parasites and pseudoparasites in wolves (Canis lupus) across continents: A comparison among Yellowstone (USA), Abruzzo (IT) and Mercantour (FR) national parks
Source: Int J Parasitol Parasites Wildl. 2019 Sep 12;10:196–206. doi: 10.1016/j.ijppaw.2019.09.002 (PMC6812024; doi:10.1016/j.ijppaw.2019.09.002)
Supplement: Multimedia component 1 [file mmc1.docx]

**Supplementary Table 1.** Characteristics of the investigated wolf packs in the three study areas

| National Park | Winter of  data collection | Packs / Individuals not attributable to specific packs | Number of contributing individuals ^a^ |
| --- | --- | --- | --- |
| PNM | 2006−2007 | Haute Tinée | 2−4 |
|  |  | Moyenne Tinée | 2 |
|  |  | Vésubie-Roya | 4−5 |
|  |  | Vésubie-Tinée | 3−5 |
|  |  | Dispersed/died/  unidentified ^c^ | 7 |
|  |  | Total | 18−23 |
| PNALM | 2006−2007 | Iorio | 6 |
|  |  | Orsara | 3 |
|  |  | Villavalelonga | 7 |
|  |  | Mainarde | 9 |
|  |  | Total | 25 |
| YNP | 2007−2008 | Slough Creek | 14 |
|  |  | Druid Peak | 16 |
|  |  | Blacktail Deer Plateau | (not constituted yet) |
|  |  | Total | 30 |
|  | 2008−2009 | Slough Creek | (disappeared) |
|  |  | Druid Peak | 16 |
|  |  | Blacktail Deer Plateau | 6−9 |
|  |  | Total | 22−25 |
| Total number of contributing individuals | | | |
| Europe |  |  | 43−48 |
| United States |  |  | 36−39 ^d^ |
| Total count |  |  | 79−87 |

^a^ PNALM: based on snow-tracking (Ciucci and Boitani, 2009; Grottoli, 2011); PNM: based on snow-tracking and non-invasive genetic sampling (Duchamp et al., 2012; ONCFS Réseau Loup/Lynx, 2007). YNP: based on daily tracking; variations are due to dispersal and death events.

^c^ Individuals either unidentified (n=1), or from which only one sample was found (n=6), as indicated by genetic analyses of mitochondrial DNA (Duchamp et al. 2012).

^d^ Six males from the Druid Peak pack joined four females from a neighbouring pack in November 2008. They established the Blacktail Deer Plateau pack (Baan et al. 2014) and are counted once.

**Supplementary Table 2.** Canid endoparasites detected in 342 wolves’ faecal samples of three different wolf populations from PNALM (2006−2007), PNM (2006−2007), and YNP (2007−2009). The total number of analysed samples (N), the number of samples that proved positive (N+), proportion (P) and corresponding 95% confidence intervals (CI) are specified. P and CI are expressed as percentages (%).

| Parasite taxa | | PNALM (N=88) | | | | | PNM (N=68) | | | | | | YNP (N=186) | | | | | | Total (N=342) | | | | | |
| --- | --- | --- | --- | --- | --- | --- | --- | --- | --- | --- | --- | --- | --- | --- | --- | --- | --- | --- | --- | --- | --- | --- | --- | --- |
|  |  | N+ | P | | 95% CI | | N+ | | P | | 95% CI | | N+ | | P | | 95% CI | | N+ | | P | | 95% CI | |
| Protozoa | *Cystoisospora* spp. ^a, b^ | 1 | 1.1 | 0.1−7.1 | | 0 | | 0 | | - | | 1 | | 0.5 | | 0.0−3.4 | | 2 | | 0.6 | | 0.1−2.3 | |  |
|  | *Sarcocystis* spp. ^c^ | 9 | 10.2 | 5.1−19.0 | | 3 | | 4.4 | | 1.1−13.2 | | 63 | | 33.9 | | 27.2−41.2 | | 75 | | 21.9 | | 17.7−26.8 | |  |
| Trematoda | *Alaria* spp. ^c^ | 2 | 2.3 | 0.4−8.7 | | 0 | | 0 | | - | | 3 | | 1.6 | | 0.4−5.0 | | 5 | | 1.5 | | 0.5−3.6 | |  |
| Cestoda | Taeniidae ^c, d^ | 14 | 15.9 | 9.3−25.6 | | 18 | | 26.5 | | 16.8−38.8 | | 125 | | 67.2 | | 59.9−73.8 | | 157 | | 45.9 | | 40.6−51.3 | |  |
| Nematoda | *Capillaria aerophila* ^b, e^ | 16 | 18.2 | 11.1−28.1 | | 0 | | 0 | | - | | 0 | | 0 | | - | | 16 | | 4.7 | | 2.8−7.6 | |  |
|  | *Capillaria boehmi* ^b^ | 71 | 80.7 | 70.6−88.0 | | 0 | | 0 | | - | | 0 | | 0 | | - | | 71 | | 20.8 | | 16.7−25.5 | |  |
|  | *Physaloptera* spp. ^c^ | 2 | 2.3 | 0.4−8.7 | | 0 | | 0 | | - | | 0 | | 0 | | - | | 2 | | 0.6 | | 0.1−2.3 | |  |
|  | *Toxascaris leonina* ^b^ | 1 | 1.1 | 0.1−7.1 | | 0 | | 0 | | - | | 30 | | 16.1 | | 11.3−22.4 | | 31 | | 9.1 | | 6.3−12.7 | |  |
|  | *Toxocara canis* ^b^ | 2 | 2.3 | 0.4−8.7 | | 0 | | 0 | | - | | 0 | | 0 | | - | | 2 | | 0.6 | | 0.1−2.3 | |  |
|  | *Trichuris vulpis* ^b^ | 0 | 0 | - | | 1 | | 1.5 | | 0.1−9.0 | | 7 | | 3.8 | | 1.7−7.9 | | 8 | | 2.34 | | 1.0−4.5 | |  |
|  | *Uncinaria* *stenocephala* ^b^ | 14 | 15.9 | 9.3−25.6 | | 1 | | 1.5 | | 0.1−9.0 | | 0 | | 0 | | - | | 15 | | 4.4 | | 2.6−7.3 | |  |
| Unidentified larvae | | 12 | 13.6 | 7.5−23.0 | | 29 | | 42.6 | | 30.9−55.2 | | 11 | | 5.9 | | 3.1−10.6 | | 52 | | 15.2 | | 11.7−19.6 | |  |
| Parasite taxa |  | 10 |  | |  | | 4 | |  | |  | | 6 | |  | |  | | 11 | |  | |  | |

^a^ Additional possible *Cystoisospora* spp. that could not be differentiated from *Eimeria* spp.: PNALM (N=2), PNM (N=5), YNP (N=1).

^b^ Parasite taxa directly transmitted (some taxa as e.g. *T. canis* might also be transmitted prenatally, transmammary or via paratenic hosts).

^c^ Obligatory, indirectly transmitted parasite taxa.

^d^ *Taenia* spp./*Echinococcus* spp. Taxa were identifiable to family level only (Bowman, 2009; Foreyt, 2001).

^e^ = *Eucoleus aerophilus*

**Supplementary Table 3.** Model selection and effects of the presence of free-ranging dogs on infection status by *Uncinaria stenocephala*. Data refer to faecal samples collected from three wolf populations: from PNALM (2006−2007), PNM (2006−2007), and YNP (2007−2009). All models were fitted with a random intercept for pack identity nested within study area, and a random intercept for winter of sample collection. Only one model is shown, as other candidate models had ∆AICc > 10. R^2^: Nagelkerke Pseudo-R^2^ (selected models only); K: number of estimable parameters; AICc: Akaike Information Criteria adjusted for small sample sizes; w: Akaike weight.

| Fixed-effects parameters | | R^2^ | | k | log likelihood | AICc | | w |  |
| --- | --- | --- | --- | --- | --- | --- | --- | --- | --- |
| Free-ranging dogs | | 0.466 | | 2 | -45.065 | 98.131 | | 1 | |
| Fixed-effect parameters | β | | SE | | 95 % confidence interval | | | |  |
|  |  |  |  |  | lower | | upper | |  |
| (intercept) | -5.505 | | 1.000 | | -7.469 | | -3.542 | |  |
| Free-ranging dogs ^a^ | 3.840 | | 1.004 | | 1.795 | | 5.886 | |  |

^a^ Reference: Free-ranging dogs absent

**Supplementary Table 4.** Model selection and effects of pack size on infection status by *Toxascaris leonina*. Data refer to faecal samples collected from three wolf population: from PNALM (2006−2007), PNM (2006−2007), and YNP (2007−2009). All models were fitted with a random intercept for pack identity nested within study area, and a random intercept for winter of sample collection. Only one model is shown, as other candidate models had ∆AICc > 10. R^2^: Nagelkerke Pseudo-R^2^ (selected models only); K: number of estimable parameters; AICc: Akaike Information Criteria adjusted for small sample sizes; ∆AICc = (AICc) – (AICc)min; w: Akaike weight.

| Fixed-effects parameters ^a^ | | | R^2^ | | K | log likelihood | AICc | | w |
| --- | --- | --- | --- | --- | --- | --- | --- | --- | --- |
| Pack size | | | 0.50 | | 2 | -83.230 | 174.461 | | 1 |
| Fixed-effect parameters | | β | | SE | | 95 % confidence interval | | | |
|  |  |  |  |  |  | lower | | upper | |
| (intercept) | | -7.230 | | 1.819 | | -10.795 | | -3.666 | |
| Pack size | 0.374 | | | 0.871 | | 0.136 | | 0.641 | |

**Supplementary data. Figure 1:** Pictures of microscopically identified wolf parasite taxa in the analysed wolf scats. A. *Isospora* sp.; B. *Sarcocystis* sp.; C. *Alaria* sp.; D. Taeniidae; E. *Capillaria aerophila*, insert shows network of ridge on shell surface; F. *Capillaria boehmi*, insert shows pitted shell surface; G. *Physaloptera* sp.; H. *Toxascaris leonina*; I. *Toxocara canis*; J. *Trichuris vulpis*; K. *Uncinaria stenocephala*. The bar in all pictures indicates 50µm, except in picture G (bar = 100µm).

A

B

C

D

E

F

G

H

I

J

K
